# Force Production and Electromyographic Activity during Different Flywheel Deadlift Exercises

**DOI:** 10.3390/sports12040095

**Published:** 2024-03-29

**Authors:** Sergio Maroto-Izquierdo, David García-López, Marco Beato, Iker J. Bautista, José L. Hernández-Davó, Javier Raya-González, Fernando Martín-Rivera

**Affiliations:** 1 i+HeALTH Strategic Research Group, Department of Health Sciences, European University Miguel de Cervantes, 47012 Valladolid, Spain; 2 Proporción A, Applied Sports Science Centre, 47015 Valladolid, Spain; 3 Department of Health Sciences, European University Miguel de Cervantes, 47012 Valladolid, Spain; 4 School of Health and Sports Sciences, University of Suffolk, Ipswich IP4 1QJ, UK; 5 Institute of Health and Wellbeing, University of Suffolk, Ipswich IP4 1QJ, UK; 6 Institute of Sport and Allied Health, University of Chichester, Chichester PO19 6PE, UK; 7 Faculty of Health Sciences, Universidad Isabel I, 09003 Burgos, Spain; 8 Faculty of Sports Science, Universidad de Extremadura, 10001 Cáceres, Spain; 9 Prevention and Health in Exercise and Sport, University of Valencia, 46010 Valencia, Spain

**Keywords:** isoinertial, eccentric, strength, hamstrings, muscle activation, team sports

## Abstract

This study aimed to characterize and compare force production and muscle activity during four flywheel deadlift exercises (bilateral [Bi] vs. unilateral [Uni]) with different loading conditions (vertical [Ver] vs. horizontal [Hor]). Twenty-three team-sport athletes underwent assessments for exercise kinetics (hand-grip force), along with surface electromyography (sEMG) of the proximal (BFProx) and medial biceps femoris (BFMed), semitendinosus (ST), and gluteus medius (GM). Mean and peak force were highest (*p* < 0.001) in Bi + Ver compared with Bi + Hor, Uni + Ver, and Uni + Hor. Although no significant differences were observed between Bi + Hor and Uni + Ver, both variants showed higher (*p* < 0.001) average force and peak eccentric force when compared with Uni + Hor. The presence of eccentric overload was only observed in the vertically loaded variants. Bi + Ver and Uni + Ver showed higher (*p* < 0.05) sEMG levels in BFProx and BFMed compared with the Uni + Hor variant. In addition, Uni + Ver registered the largest GM and ST sEMG values. In conclusion, the vertical variants of the flywheel deadlift exercise led to higher muscle force production and sEMG compared with their horizontal counterparts. Both Bi + Ver and Uni + Ver may be effective in promoting an increase in hamstring muscles activity and muscle force at long muscle length, while the Uni + Ver variant may be more effective in promoting GM and ST involvement.

## 1. Introduction

It is well-known that musculoskeletal trauma occurs because the load imposed on body tissue exceeds the tissue’s load tolerance. During sport-specific demanding eccentric actions, such as the late swing phase of high-speed running or landing from a jump [1], large loads are often placed on athletes’ body, which in turn may result in severe non-contact injuries (e.g., ACLs and muscle injuries). Modifiable risk factors for non-contact injuries in team sports athletes include low levels of strength and low rates of force development in hamstring muscles and hip stabilizer muscles [2], low hamstring muscle endurance [3], reduced lumbo-pelvic stability [4], lack of positive muscle architecture adaptations (i.e., long fascicle length and wide fascicle angles) [5,6], longer electromechanical delay of the hamstrings (i.e., intramuscular coordination and neuromuscular fatigue), and altered intermuscular coordination between hamstring muscles and between hamstring and hip abductor muscles [3]. Thus, a variety of strategies are being used to modify these risk factors in an attempt to prevent non-contact injuries [7,8,9].

High-intensity resistance training has been found to be one of the most effective strategies at improving muscular strength and thereby reducing the risk of acute and overuse sport injuries [10]. However, exercise intensity during resistance training is generally determined by an athlete’s maximal concentric muscle strength (i.e., the ability to lift rather than lower the load) [11], thus, due to the well-described force–velocity characteristics of muscles, providing an underloaded eccentric stimulus [12]. In addition, the high presence of eccentric actions in team sports, such as decelerations, changes of direction, and sprints [9], the high injury rate registered during decelerative movements [13], and the necessity to provide a greater and more complete neuromuscular stimulus together with the need to cause more favorable structural adaptations [7], have generated growing interest regarding eccentric exercises among strength and conditioning (S&C) coaches and medical staff. Therefore, exercises involving eccentric fascicle behavior or requiring rapid force production at longer muscle lengths might be beneficial for modifying non-contact injury risk factors [13]. However, it is well-established that the closer the mechanical specificity between exercises and outcome performance, the greater the transfer of enhanced capacity [14]. This specificity should encompass a variety of factors, including movement pattern and velocity, type of contraction, exercise intensity (strength vs. endurance needs), stability requirements, direction of force vector, joint angular momentum, range of motion, and other relevant capacities [15]. Consequently, due to the nonspecific nature of eccentric-only training, eccentrically accentuated loading has emerged as the preferred mode of exercise [16]. This preference arises because it yields greater neuromuscular effects by utilizing higher absolute intensities during lengthening contractions while also providing more specific force demands through the incorporation of the stretch–shortening cycle (SSC) [17].

The scientific literature identifies several resistance training methods that enable practitioners to emphasize eccentric contractions, sometimes achieving eccentric overload. These methods include flywheel training [18], accentuated eccentric loading [16], and high-speed plyometric training [19]. Among them, flywheel resistance training stands out for its greater versatility and high muscular demands during both concentric and eccentric contractions [20,21], positioning flywheel training as a valid alternative to traditional resistance training for enhancing muscular strength, power, and jump performance with untrained and trained populations [18,21]. Despite the primary limitations associated with flywheel technology, including the requirement for maximal concentric contractions to generate momentum in the flywheel, which then facilitates the production of significant active braking forces during the brief terminal phase of the eccentric contraction [12] and challenges in monitoring and tailoring training volumes and intensities to meet individual needs [20], this technology offers the unique advantage of enabling sport-specific exercises [21]. Its independence from gravity allows for its application in both predominantly horizontal and vertical movements [22]. In addition, due to the unilateral nature of several sport-specific tasks, unilateral exercises result in a more specific stimulus compared with traditional bilateral lifting [15], leading to a superior transfer to athletic performance and injury prevention [22]. Therefore, strength exercises that mimic as closely as possible athletic movement patterns, such as the single-leg deadlift for running mechanics, that apply larger moment arms of the hamstrings around the hip compared with the knee [5], leading to high eccentric force production when the muscle–tendon unit reaches its peak length (resulting in higher fascicle length changes during exercise), have been implemented in resistance training programs [5]. Indeed, unilateral flywheel training, which provides predominantly horizontal resistance more akin to the demands of sports [22], has been linked to greater improvements in sport-specific tasks, including change of direction and horizontal jumping, compared with bilateral training [22,23]. Similarly, unilateral flywheel training has been associated with a lower injury occurrence in team-sport athletes [24]. However, more high-quality studies are needed to further elucidate the superiority of horizontally eccentric overloaded exercises in training-related variables associated with performance and injury prevention.

To provide a training stimulus that enhances tissue tolerance while improving performance in team-sport athletes, some studies have analyzed the surface electromyographic activity (sEMG) of the hamstring muscles, comparing various flywheel exercises [25,26,27,28]. However, to the best of our knowledge, the effects of a more demanding unilateral sport-specific (i.e., movement that mimics a recurring movement pattern) and injury-specific (i.e., developing high eccentric force at long muscle–tendon unit length) exercise, such as the flywheel single-leg deadlift, on sEMG of the hip stabilizer, hip extensor, and knee flexor muscles and its kinetic characteristics (e.g., eccentric overload in terms of force) in comparison with its bilateral variant remain unknown. Similarly, it is unidentified whether these variables are affected using either predominantly horizontal or vertical loading strategies. Therefore, this study aimed to analyze and compare force production and the sEMG of the biceps femoris (proximally and medially), semitendinosus, and gluteus medius muscles during the flywheel deadlift exercise in different force vectors (horizontal and vertical) and during different situations (unilateral and bilateral) in team-sport athletes. Given the evidence that more demanding unilateral sport-specific exercises lead to a superior transfer to athletic performance and injury prevention, it was hypothesized that unilateral variants of the flywheel deadlift exercise would evoke greater muscle activation of the hip stabilizing musculature and require higher muscle force compared with the amount of force produced by a single limb during bilateral variants. In addition, we hypothesize that vertical flywheel exercise variants are those in which we can achieve greater activation of the biceps femoris and semitendinosus muscles due to the large hip flexion and large moment arm of the hamstrings around the hip.

## 2. Materials and Methods

### 2.1. Study Design

This within-subjects (repeated measures) experimental study consisted of two familiarization sessions and two experimental sessions. The first two sessions were used to familiarize participants with the flywheel deadlift exercise in both unilateral and bilateral situations and with both vertical and horizontal loading exercise variants. During the third and fourth visit to the laboratory (i.e., experimental sessions), participants performed the horizontally and vertically loaded flywheel deadlift exercise in both unilateral and bilateral situations in a counterbalanced and randomized order. Proximally (BF_Prox_) and medially biceps femoris (BF_Med_), semitendinosus (ST) and gluteus medius (GM) sEMG, and force production at grip level were measured during exercise. This study was conducted during the competitive period between November and December. The familiarization and evaluation sessions were scheduled on days furthest from competition.

### 2.2. Participants

Twenty-three healthy team-sport athletes (mean ± SD; 21.5 ± 2.5 years, 172.5 ± 8.0 cm, 70.4 ± 9.9 kg; eleven females: 21.5 ± 2.5 years, 166.6 ± 5.4 cm, and 62.0 ± 2.9 kg; and twelve males: 21.6 ± 2.5 years, 178.0 ± 6.2 cm, and 78.2 ± 7.1 kg) with no history of lower-limb orthopedic injuries volunteered to participate. Participants were engaged in 8–12 h per week of sport-specific training and participated at professional or semiprofessional levels in soccer (*n* = 10), basketball (*n* = 7), and handball (*n* = 6), and regularly performed strength training exercises. All of them had at least one year of experience with flywheel training. The athletes enrolled were informed of the purposes and risks involved in the study before giving their informed written consent to participate. All participants completed all the protocols, including two familiarization sessions and, one week later, the prescribed two testing sessions separated by 72 h. Volunteers visited the laboratory at the same time and under the same conditions on each testing day. Health, physical activity, and hydration status were assessed (Physical Activity Readiness Questionnaire, International Physical Activity Questionnaire, and Beverage Intake Questionnaire). Moreover, participants recorded and then maintained their sleeping, eating, and drinking habits in the 48 h prior to each testing session. Stimulant consumption was recorded on the day of the first testing session and replicated on the second testing day. In addition, participants were not able to exercise strenuously within 72 h prior to testing sessions. These study procedures were in accordance with the principles of the Declaration of Helsinki and were approved by the local Institutional Review Board (H1421157445503).

### 2.3. Procedures

**Figure 1:**
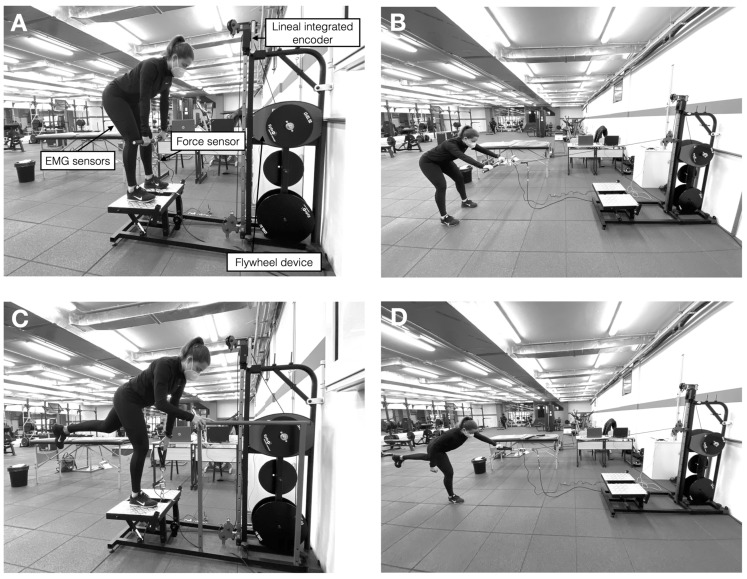
Experimental set-up for the vertically loaded bilateral deadlift (**A**), the horizontally loaded bilateral deadlift (**B**), the vertically loaded single-leg deadlift (**C**), and the horizontally loaded single-leg deadlift (**D**) on the flywheel device used.

The flywheel deadlift exercise was performed in four different situations: (1) vertically loaded in a bilateral situation (Bi + Ver), (2) vertically loaded in a unilateral situation (Uni + Ver), (3) horizontally loaded in a bilateral situation (Bi + Hor), and (4) horizontally loaded in a unilateral situation (Uni + Hor). All these exercise variants were performed randomly in a flywheel device (Epte Inertial Concept, Ionclionis and Deionic, LAlcúdia, Valencia, Spain). In the case of the vertically loaded variants, participants performed the deadlift exercise by standing upright on the flywheel device platform (Figure 1A,B) while holding the device’s barbell (Bi + Ver) or hand grip with the contralateral hand (Uni + Ver). When performing the Bi + Ver variant, participants were instructed to slightly blend the knees and point the toes straight ahead, with a straight back and a retracted scapula with pelvic anteversion that must be maintained during exercise. Participants were required to perform a gently eccentric contraction by flexing the hip allowing the torso and barbell to lower to the anterior tuberosity of the tibia height, where the upper body was almost horizontal (90° hip flexion; Figure 1A) and where a maximal eccentric contraction occurred to break the movement, just before extending their hip and returning to an upright position (0° hip flexion) after a maximal concentric contraction. Participants were instructed to keep the barbell close to the body. When the Uni + Ver variant was used, participants were instructed to slightly blend the knee of the dominant leg, point the toes straight ahead, and transfer their weight onto this leg to perform the exercise, maintaining the same position described above. The participant’s contralateral limb was kept extended and at no time contacted the floor during the exercise. All participants were instructed to avoid hip abduction and adduction of the non-exercised leg, keeping the toes of the non-exercised leg pointing downwards. Participants were instructed to perform the exercise holding the grip with the contralateral hand, while they were allowed to place the ipsilateral hand on a fixed point just to provide stability to the movement (Figure 1B). Similarly, in the case of the horizontally loaded variants, participants performed the deadlift exercise by standing on the floor in front of the flywheel device (Figure 1C,D) at approximately 2 m (so that the maximum length of the flywheel cable coincides with the end of the range of motion of the concentric phase) while holding the device’s barbell (Bi + Hor) or hand grip (Uni + Hor). In this sense, the movement began in a fully upright position, with the hands at chest height, placing the barbell or the grip 5 cm from the participant’s chest. Participants were then instructed to perform a gently eccentric contraction until they reached 90° hip flexion, at which point they were requested to perform a maximal eccentric contraction to break the movement and to claim the fully upright position (0° hip flexion) through a maximal concentric contraction. The height of the cable and the distance to the flywheel device were individually regulated for each participant. The technical characteristics described for both vertically loaded variants were also maintained. In the case of Uni + Hor, participants were allowed to place the ipsilateral hand on a fixed point to provide stability to the movement.

During the first familiarization session, each participant was shown a demonstration of the correct technique for all vertically loaded flywheel deadlift variants by one of the investigators and then performed several practice repetitions of the exercise with different loads, starting with lighter inertial loads (0.03 kg·m^2^) and progressively increasing to heavier ones (0.0662 kg·m^2^). These initial exercises also served as an introduction to exercise intensity [29]. The second familiarization session was performed at least three days after the first familiarization session to minimize the influence of delayed-onset muscle soreness [30]. On this second session, participants were instructed to perform properly both bilateral and unilateral variants of the horizontally loaded flywheel deadlift exercise with progressive loads by one of the investigators. During both sessions, they performed at least 3 sets of 6–8 repetitions with the testing load (0.0662 kg·m^2^) on each exercise variant (bilateral and unilateral). All participants were instructed to perform a maximal concentric contraction and to decelerate in the final third of the movement. During both familiarization sessions, visual feedback on the peak power of each contraction was provided to the participants to ensure correct technique and the development of greater eccentric peak power.

One week later, 2 testing sessions 48 h apart were performed to compare muscle activity and force production at hand-grip level between the 4 selected flywheel deadlift variants. In a counterbalanced and randomized order, participants performed a total of 4 sets of either vertically or horizontally oriented flywheel deadlift exercises in both bilateral and unilateral conditions in both testing sessions. The inertial load used was 0.0662 kg·m^2^, which was chosen because it allowed for the highest mean concentric power compared with other loads tested such as 0.0472, 0.0662, and 0.1040 kg·m^2^ in a previous pilot study, where we compared the mean concentric power developed in the four variations of the flywheel deadlift exercise. Additionally, it was chosen based on previous studies where the most common moment of inertial employed was between 0.05 and 0.145 kg·m^2^ [29].

During testing, two sets of each exercise were performed. Each set was composed of 8 repetitions. The first repetition was used to induce momentum in the flywheel system; meanwhile, during the second repetition, participants were asked to perform maximal concentric action throughout the full range of motion (established between feet and the anterior superior iliac spine, in case of vertically oriented exercises; and between 0° hip flexion until 90° hip flexion in the case of horizontally oriented exercises) to apply a short and concentrated eccentric contraction in the last third of the range of motion, as previously described. The flywheel system used an integrated linear quadrature encoder to detect the change of each phase upon altering the input channel designated for each phase of the movement (Epte encoder, Ionclionis and Deionic, LAlcúdia, Valencia, Spain). This encoder was incorporated into a secondary pulley through which the device’s cable was threaded, aiming to detect the linear movement of the cable and the direction of movement, functioning similarly to a traditional linear position transducer [31]. The encoder collected data with a resolution of 48 points per revolution (equivalent to 4.8 mm) and at a frequency of 1000 Hz [31]. In this study, the encoder-generated data encompassed both concentric and eccentric peak power and was leveraged to provide participants with visual feedback. This approach was designed not only to motivate participants to exert maximum effort in each concentric muscle contraction but also to ensure proper technical execution and confirm the attainment of eccentric overload in terms of peak power on each repetition. The protocol was replicated 48 h later in the other force vector that was not used in the first session.

Each testing session was preceded by a comprehensive task-specific warm-up designed to impact the musculature most closely related to the deadlift exercise. It consisted of 5 min of cycling followed by 5 min of a dynamic stretching protocol (e.g., forward leg swings, ankle dorsi- and plantar-flexion, side leg swings, high knees, heel flicks, squats, lunges, and bodyweight single leg deadlifts) [32]. Each exercise was performed for 20 s, and the entire set was repeated twice. Then, two sets of eight continuous unloaded single-leg bodyweight deadlift interspersed by 30 s at 1/1 tempo were performed with each leg. Finally, each participant performed 4 maximal voluntary isometric contractions (2 contractions in each bilateral or unilateral condition). The maximal voluntary isometric contraction was performed at the end of the range of motion of each exercise, where the upper body was almost horizontal (90° hip flexion) holding the barbell or the handgrip depending on the exercise variant.

### 2.4. Data Collection

Surface electromyogram (sEMG) amplitudes were measured from BF_Prox_, BF_Med,_ ST, and GM of the dominant leg using a wireless dual sEMG system (EMG MuscleLab, ML6000, Ergotest Innovation AS, Bjønnveien, Noruega). A bipolar configuration of two Ag/AgCl self-adhesive electrodes (1 cm inter-electrode distance; White Sensor WS, 79 mm^2^, Ambu, Ballerup, Denmark) was placed in approximate alignment with the muscle fibers, as per the SENIAM guidelines [33]. Proximal and distal electrode pairs were placed at 25% and 50% on the line between the ischial tuberosity and the lateral epicondyle of the tibia for the proximal and medial portions of the biceps femoris, respectively, since muscle activation is heterogeneous through this muscle [34]; at 50% on the line between the ischial tuberosity and the medial epicondyle of the tibia over the belly of the semitendinosus muscle; and at 50% on the line from the iliac crest to the trochanter for the gluteus medius muscle. The skin beneath the electrodes was shaved, abraded, and cleaned with alcohol to reduce inter-electrode resistance prior to each testing session. Correct placement was identified by palpation and confirmed by visual observation of the sEMG signal during voluntary contractions. Electrode placement was marked on the skin with permanent marker and replicated in the second testing session. sEMG data were collected synchronously with the torque data during exercise, and amplified (×1000) and filtered using a 20–500 Hz band-pass filter, and converted online to root mean square sEMG (sEMG_RMS_) with a 100 ms symmetrical moving average window [5]. For analysis, the maximum activation recorded throughout the full range of motion during the entire exercise (sEMG_peak_) for each muscle analyzed was collected during exercise and used to normalize the sEMG data [35].

Muscle force produced by the participant at grip level during each repetition was assessed with a force sensor (Muscle Lab, Electro-Extensionisometric cell, Ergotest Innovation AS, Bjønnveien, Norway). This force sensor was placed between the handle grip and the distal part of the flywheel device’s cable to avoid dissonances between what happened at ground level and what happened at participant level. Force signals were sampled at 1000 Hz. The primary outcomes utilized in the statistical analyses were concentric and eccentric peak force (i.e., the highest projection on the force–time curve for each concentric and eccentric contraction) and average force (i.e., the mean force exerted throughout the entire repetition) of the repetition that demonstrated the highest concentric and eccentric peak force. In addition, linear velocity was controlled with a linear encoder (1000 Hz sampling rate) incorporated by the flywheel device. Force and sEMG data were synchronized and recorded by a data synchronization unit (MuscleLab ML6000 DSU, Ergotest Innovation AS, Bjønnveien, Noruega).

### 2.5. Statistical Analyses

All statistical analyses were performed using the Jamovi software package (The Jamovi Project, v.1.6.23.0; downloadable at https://www.jamovi.org accessed on 28 March 2024). Normality was checked by the Shapiro–Wilk normality test. Then, a repeated measures linear-mixed model fitted with a restricted maximum likelihood method and unstructured covariates was used to compare outcomes between exercises (bilateral and unilateral) and force vector (vertical and horizontal). The main outcomes used in statistical analyses were peak and average force of the entire repetition, and sEMG_RMS_ and peak normalized sEMG activation for the BF_Prox_, BF_Med_, ST, and GM muscles. The level of significance for all tests was set at *α* = 0.05. Mean, standard error (SE), and t value were reported for all statistical analyses. In addition, the descriptive values of mean, upper limit, and lower limit of each outcome (force and muscle activity) were represented graphically for each exercise. These graphical representations were created using MATLAB 9.12 (R2022a, Natick, MA, USA: The MathWorks Inc., 2022) for the treatment of the raw signals, the interpolation and initial preparation of the graphs, and, subsequently, to represent them. For this purpose, the raw data of the best repetition of each exercise in terms of force (i.e., the highest mean force of each participant and exercise variant) were interpolated to 1D (101 data).

Sample size was estimated *a priori* for ANOVA repeated measures using G*power (G*Power 3.1.9.2, Heinrich Heine Universitat Dusseldorf, Dusseldorf, Germany; http://www.gpower.hhu.de/ accessed on 29 March 2024). The effect size was computed using the means and between-subject SDs from a previously published study [33] that investigated biceps femoris muscle activity during bilateral and during unilateral deadlift exercise of the dominant leg. The means and SDs were 60.2 ± 25.2% and 68.9 ± 24.8% of maximal voluntary isometric contraction, respectively. The mean difference and average SD were therefore 8.7% and 25.0%, respectively, resulting in a Cohens *d_z_* effect size of 0.70, which can be classified as equivalent to f = 0.3 (moderate). The average SD was used to compute the effect size. Alpha was set at 5%, while power was set at 80% (1 − *β*). The estimated sample size was 17 participants (actual power = 0.814), but, considering possible dropouts, we enrolled 23 participants in this study.

## 3. Results

**Figure 2:**
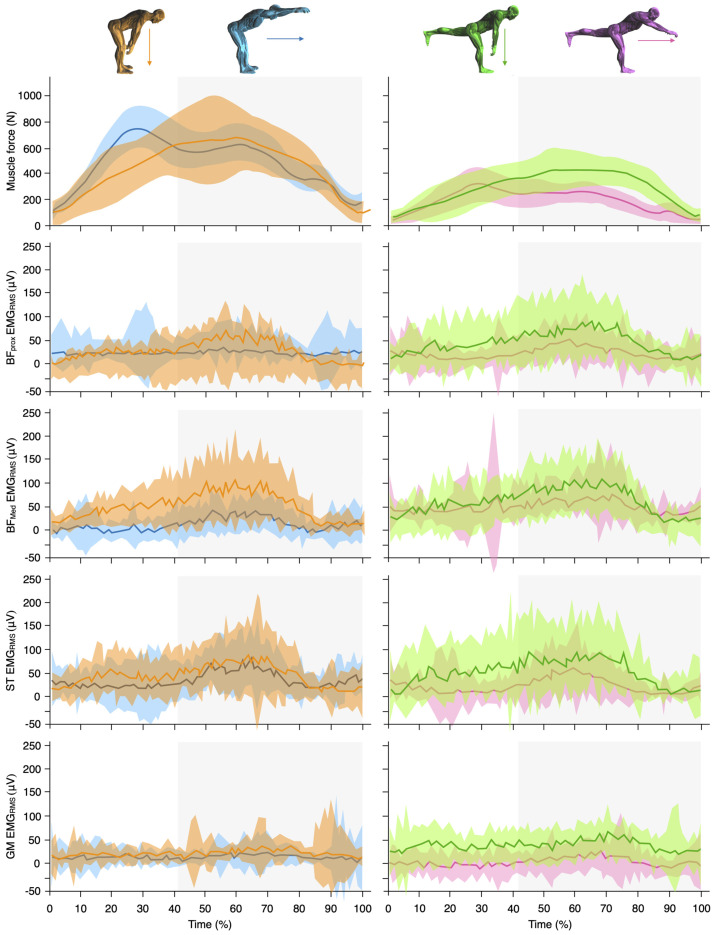
Force production (N) at hand-grip level (**first row**), biceps femoris muscle activity at proximal (**second row**) and medial level (**third row**), semitendinosus muscle activity (**fourth row**) and gluteus medius muscle activity (**fifth row**) during the bilateral vertically loaded (blue), bilateral horizontally loaded (orange), unilateral vertically loaded (green), and unilateral horizontally loaded (pink) flywheel deadlift exercise. Each panel displays the group mean (solid line) ± mean upper and lower limits (shaded band) during the total duration of a single repetition. The light grey area reflects the mean eccentric contraction phase for the bilateral (~41%) and unilateral (~43%) flywheel deadlift exercise variants.

Regarding force production, significant effects were shown for exercise condition (i.e., bilateral vs. unilateral condition: *p* < 0.001, F = 67.6; and *p* < 0.001, F = 37.8) and loading condition (i.e., vertically vs. horizontally loading condition: *p* < 0.001, F = 201.7; *p* < 0.001, F = 61.5; and *p* < 0.001, F = 45.9) for both average, concentric, and eccentric peak force, respectively. In addition, significant interactions were observed between exercise condition and loading condition (*p* < 0.001, F = 32.7; and *p* = 0.013, F = 7.4). As shown in Table 1 and Figure 2, the Bi + Ver variant manifested higher (*p* < 0.001) average force production compared with the other three exercise variants (mean [%, SE and t]: 221.8 N [85.5%, SE = 17.3, t = 12.8] when compared with Bi + Hor; 182.7 N [61.1%, SE = 21.5, t = 8.5] when compared with Uni + Ver; and 295.4 N [160.9%, SE = 20.8, t = 14.2] when compared with Uni + Hor). The Bi + Hor and Uni + Ver conditions did not show significant differences between them compared with the Uni + Hor condition (73.6 N, 40.6%, *p* < 0.001, SE = 14.3, t = 5.2; and 112.7 N, 62.0%, *p* < 0.001, SE = 12.6, t = 9.0, respectively). Regarding eccentric peak force, similar results were observed. The Bi + Ver variant showed higher (*p* < 0.001) values compared with the Bi + Hor (164 N, 27.7%, SE = 59.1, t = 5.2), Uni + Ver (259 N, 52.7%, SE = 51.6, t = 5.0), and Uni + Hor (490 N, 184.2%, SE = 59.2, t = 7.1) variants. As spotted in average force, the Bi + Hor and Uni + Ver conditions did not show significant differences between them in eccentric peak force compared with the Uni + Hor condition (112 N, 35.6%, *p* < 0.001, SE = 24.7, t = 4.5; and 161 N, 51.8%, *p* < 0.001, SE = 19.2, t = 8.4, respectively). Regarding concentric peak force, both Bi + Ver and Bi + Hor variants showed similar higher (*p* < 0.001) values compared with Uni + Ver (268 N, 71.5%, *p* < 0.001, SE = 54.3, t = 5.5; and 321 N, 85.6%, *p* < 0.001, SE = 28.6, t = 8.0, respectively) and Uni + Hor (317 N, 97.2%, *p* < 0.001, SE = 24.3, t = 6.1; and 370 N, 113.5%, *p* < 0.001, SE = 22.6, t = 8.9, respectively) variants. However, the Uni + Ver and Uni + Hor conditions did not show significant differences between them in concentric peak force. Ultimately, only the vertical variants were shown to develop eccentric overload in terms of peak force, with an average overload of 18% for the Bi + Ver variant and 32% for the Uni + Ver variant.

Individual analysis of sEMG_RMS_ and sEMG_peak_ of each muscle was performed (Table 1 and Figure 2). Regarding BF_Prox_, significant effects were shown for loading condition (i.e., vertically vs. horizontally loading condition: *p* = 0.012, F = 7.5,) for sEMG_RMS_. As shown in Table 1, the Uni + Ver variant showed higher BF_Prox_ sEMG_RMS_ (5.2%, *p* = 0.007, SE = 1.4, t = 3.7) and sEMG_peak_ (63.9 μV, *p* = 0.05, SE = 23.3, t = 2.7) values when compared with Uni + Hor. When BF_Med_ was analyzed, a significant effect was observed for loading condition (*p* < 0.001, F = 19.0) for sEMG_RMS_. In addition, higher sEMG_RMS_ was registered in Uni + Ver (5.7%, *p* < 0.001, SE = 1.2, t = 5.0) and Bi + Ver (4.4%, *p* = 0.014, SE = 1.3, t = 3.4) when compared with the Uni + Hor variant. Moreover, both vertical variants showed higher sEMG_peak_ values than Bi + Hor (Bi + Ver: 89.0 μV, *p* = 0.01, SE = 25.1, t = 3.5; and Uni + Ver: 125.3 μV, *p* > 0.001, SE = 23.6, t = 5.3). Regarding ST, significant effects were shown for exercise condition (*p* = 0.005, F = 9.7) and loading condition (*p* < 0.001, F = 18.3) for sEMG_peak_. However, a significant interaction between exercise condition and loading condition was only observed for sEMG_RMS_ (*p* = 0.002, F = 12.3). As shown in Table 1, the Uni + Ver variant showed higher sEMG_RMS_ (5.9%, *p* = 0.024, SE = 1.9, t = 3.1) and sEMG_peak_ (97.2 μV, *p* = 0.047, SE = 36.8, t = 3.6) values when compared with Uni + Hor, and higher sEMG_peak_ in comparison with the Bi + Hor variant (163.5 μV, *p* < 0.001, SE = 31.6, t = 5.2). Similarly, the Bi + Hor variant demonstrated higher sEMG_RMS_ (5.3%, *p* = 0.003, SE = 1.3, t = 4.1) and sEMG_peak_ (66.2 μV, *p* = 0.008, SE = 18.2, t = 3.6) than the Uni + Hor variant. Moreover, the Bi + Ver showed greater sEMG_peak_ (112.6 μV, *p* = 0.005, SE = 29.3, t = 3.8) values compared with Bi + Hor. Finally, regarding GM, significant effects were shown for exercise condition (sEMG_peak_: *p* = 0.043, F = 4.6) and loading condition (sEMG_RMS_: *p* = 0.05, F = 4.4). No significant differences were observed between exercise variants in the GM sEMG_RMS_. Only Uni + Ver demonstrated higher sEMG_peak_ (99.5 μV, *p* = 0.014, SE = 29.6, t = 3.4) when compared with the Bi + Hor variant.

## 4. Discussion

This study aimed to analyze and compare force production at grip level and the sEMG of the biceps femoris (proximally and medially), semitendinosus, and gluteus medius muscles during the flywheel deadlift exercise performed in different force vectors (horizontal and vertical) and during different situations (unilateral and bilateral) in team-sport athletes. This is the first study to compare different exercise variants of the deadlift exercise performed in a flywheel device in terms of force and sEMG. Vertical flywheel deadlift exercise variants have been shown to evidence the highest mean and peak force absolute values. Specifically, the Bi + Ver variant registered higher average and peak force productions compared with the rest of the exercises analyzed. Similarly, the Uni + Ver variant showed higher mean and peak force productions than the Uni + Hor variant. Additionally, the vertical variants (Bi + Ver and Uni + Ver) were the only variants in which higher eccentric peak forces were recorded compared with concentric peak forces. Regarding muscle activation, the Uni + Ver variant registered the highest sEMG_RMS_ and sEMG_peak_ values, showing significant differences with respect to the horizontally loaded variants. For all these reasons, vertical variants of the flywheel deadlift exercise showed greater neuromuscular demands. Although there were no significant differences in sEMG outcomes between the two vertically loaded variants, the use of the Uni + Ver exercise could offer several advantages because it could be used to reduce muscle asymmetries between limbs and could be used for enhancing muscle performance in unilateral sport-specific tasks. In any case, both vertically loading variants developed higher force values at long muscle–tendon unit length, resulting in an optimal stimulus for hamstring muscles [7,9].

Neuromuscular function improvements, including increases in muscle force and an optimized muscular activation pattern, have been proposed as a fundamental strategy for injury prevention purposes [5,7,13]. Despite several studies analyzing and comparing force production and sEMG activity during different traditional strength exercises targeting hamstring muscles having been published [7,34], research assessing muscle force and activity during flywheel exercises mimicking hamstring injury mechanisms (e.g., a movement with high force demands at long muscle length) is scarce. Hip dominant exercises such as the flywheel deadlift are often used for hamstring injury prevention, in the rehabilitation process, and to enhance performance [12]. Reasons for the popularity of the deadlift exercise include the larger moment arms of the hamstrings around the hip compared with the knee [5], which results in a larger muscle–tendon unit and hence potentially fascicle length changes (leading to a high eccentric force production when the muscle–tendon unit reaches its peak length) during the deadlift exercise and its variants (e.g., single-leg deadlift) compared with more knee-dominant exercises [5]. In fact, a large body of research has shown that the fascicles work predominantly isometrically during elastic muscle functioning (i.e., when the series elastic components of the muscle stretch and recoil) since this optimally facilitates the storage and reuse of elastic energy [36], such as during the late swing phase of high-speed running [8]. Furthermore, given the high vertical ground reaction forces that occur during forward horizontal displacements such as high-speed running, and the external forces acting on the hamstrings during the late swing phase of running [37], the vertically loaded unilateral deadlift exercise has been postulated as an optimal exercise to stimulate the hamstring muscles with a high degree of specificity [5]. It is also known that many sport non-contact injuries occur in scenarios where high eccentric forces are applied at high speed (e.g., late swing phase of running or landing from a jump) [1]. This probably puts the hamstring muscles at the limit of their load capacity and hence they likely function isometrically at an angle close to their optimum fascicle length because this is where they can produce maximum protecting force [8]. Therefore, providing a stimulus of high specificity, with a high neuromuscular demand for the hamstrings (i.e., maximum concentric contraction and an accentuated eccentric action) in which eccentric force is applied at high speed, as it occurs during the flywheel deadlift, can be a model strategy for optimizing resistance training and injury prevention.

This is the first study to analyze force production during different deadlift variants with flywheel devices. The results of our study showed that the vertical loading variants generated higher absolute values of peak and average force production than the horizontal variants (Figure 2 and Table 1). Similarly, significant differences were found in the peak and mean force produced during the bilateral variants compared with the unilateral variants. Therefore, the Bi + Ver variant was the one that showed the highest absolute values of peak and mean force production. Despite this, the peak and mean force produced only by the trained leg during the unilateral variants (Uni + Ver and Uni + Hor) proved to be greater than the force produced relatively by each limb during the bilateral variants in addition to providing a more specific force production given the unilateral nature of many sports actions, as well as a greater proprioceptive demand [33]. In any case, this is the first study in which forces have been compared between bilateral and unilateral conditions and between vertical and horizontal loading variants of the deadlift exercise (Table 1), so more studies are required to know the specific effects of a training program. However, the results of this study seem to indicate that vertical deadlift exercise variants provide a more demanding neuromuscular stimulus compared with horizontal variants (i.e., demanding higher amounts of average and peak force), although horizontal variants of the deadlift may seem more specific given the large horizontal component of displacement in sports. Indeed, several studies have highlighted the benefits of horizontal exercises performed with flywheel devices [22,23]. Regarding force production, the vertical flywheel deadlift variants are more specific to unilateral sport-specific skills such as sprinting, since the greatest ground reactive force records registered in those actions in which the hamstring muscles apply large amounts of force at high muscle–tendon unit length are of vertical nature (e.g., late swing of high-speed running) [1,38]. Regarding the application of force throughout the range of motion, it is noteworthy that the vertical variants exhibit a tendency to generate higher force values towards the end of the repetition, where the musculature is more elongated (Figure 2). Indeed, only the vertical variants (Bi + Ver and Uni + Ver) recorded eccentric overload, i.e., higher eccentric peak force compared with concentric peak force (Table 1). This may be due to the fact that the kinetic energy dissipated during the horizontal variants is greater, requiring a larger range of movement and a longer eccentric–concentric transition time. Additionally, the observed differences between the vertical and horizontal vectors in the flywheel deadlift exercise may stem from the influence of gravity. For instance, Sjöberg et al. [39] showed that gravity significantly impacts exercises performed in the vertical plane compared with those in the horizontal when utilizing a flywheel device. In vertically loaded exercises like the squat, more than half of the load is provided by body weight, whereas, in horizontally loaded exercises such as the leg press exercise, the flywheel itself furnishes the entire load. This distinction underscores the unique biomechanical demands of exercises across different planes and force vectors and may also explain the variations in force production that we observed in our study. This does not mean that it is a worse variant, it is simply a variant that should be used at different season periods or microcycle moments as it implies lower neuromuscular demands (for example, closer to competition in team sports athletes) [40].

Regarding muscle activity, the results of this study showed similar muscle activation between the analyzed muscles of the hamstring complex regardless of exercise variant (bilateral vs. unilateral and horizontal vs. vertical) used, with both BF and ST showing a similar mean sEMG activity (e.g., 27–33% of maximum, Table 1). Similar to previous studies where high-density sEMG was used [41], no between-muscle differences were found between muscles during any flywheel deadlift variants. Based on our results, these exercises should be used when balanced activation of ST and BF muscles is of interest. This, in turn, suggests that ST-BF muscle selectivity cannot always be predicted based solely on the specificity of the exercise or hip- or knee-dominant nature of the exercise and may be affected by different neural control strategies in the eccentric and concentric contractions. These results are not in line with a recent systematic review that concluded that the deadlift exercise led to slightly greater ST than BF muscle activation [42]. These controversial results may be explained by methodological differences. On the one hand, the external load used in the included studies varied from 50% of two repetition maximum (RM) load to 85% of one RM. On the other hand, some included studies did not use a normalized (e.g., respect to MVIC test) sEMG value. Further, differences in muscle activity requirements between traditional and flywheel resistance exercises have been previously reported [43] and cannot be therefore discarded as a reason for these different results. Therefore, and based on our results, there is no flywheel deadlift variant that leads to a preferential muscle activation within the hamstring muscle complex. However, the present study’s results highlight the influence of loading condition on hamstring and gluteus sEMG activity, with vertical loading variants demanding higher sEMG activity in all the muscles analyzed (see Table 1), which was also accompanied with greater average and peak forces, as abovementioned. This could entail potential implications for training prescriptions aiming to reduce hamstring injury risk by reducing some assumed risk factors. It can be hypothesized that the use of flywheel deadlift vertical variations may lead to greater strength gains, which have been proposed as important factors in reducing hamstring injuries [2]. In addition, due to the intrinsic characteristics of flywheel exercises, force requirements are specially increased at long muscle lengths, which may lead to greater improvements in eccentric force at this specific muscle length and joint angle, together with significant increases in fascicle length, both being important factors in reducing hamstring injury risk [2,5,6,7].

Muscle activation has been considered a key determinant in training-induced muscle hypertrophy [7]. Diamant and colleagues [33] observed higher BF and GM activation during the unilateral deadlift when compared with the bilateral variant using free-weights, thus concluding that unilateral deadlift is preferable in training the BF and GM. In the present study, no differences in sEMG activity were found when comparing bilateral vs. unilateral flywheel exercise within the same deadlift loading condition (vertical and horizontal). However, despite sEMG activity not reaching a statistical difference when comparing unilateral vs. bilateral conditions, there was a trend for greater peak sEMG activity during unilateral variations. In addition, with the relatively greater peak forces reached by the training leg during Uni + Ver, the slightly greater sEMG activity found leads to recommend this exercise variant as an applicable tool for hamstring injury prevention purposes, supporting the perspective proposed by Diamant and coworkers [33]. As hamstring injury mechanisms include the sudden activation of the hamstrings as they lengthen [6,44], the use of this exercise may be optimal as a preventive tool, not only because of being a movement pattern similar to injury mechanisms but also by emphasizing eccentric force production due to flywheel exercise characteristics [9]. Due to the technical requirements implicit in flywheel technology, participants were required to perform the braking action at a specific moment at the end of the range of motion, just at the point where the muscle reaches its maximum elongation, therefore producing tension in a situation where the fascicles work predominantly isometrically during elastic muscle functioning, facilitating the storage and reuse of elastic energy, and therefore eliciting a higher and different activation pattern compared with shortening [5,6]. However, as muscle activation during different exercises is highly heterogeneous [7], it is most likely possible that hamstring injury prevention programs benefit from the inclusion of several exercises differing in joint patterns (hip vs. knee dominant) and number of limbs involved (bilateral vs. unilateral).

This study presents some limitations that should be considered when interpreting the findings. First, we compared various deadlift exercise variants performed on a flywheel device focused on force and sEMG metrics; however, we did not examine kinematic outputs during the concentric and eccentric phases. Consequently, further research is necessary to elucidate the power inertial loads associated with different deadlift variations. Additionally, complementing kinetic analyses with kinematic variables would yield more comprehensive insights, facilitating the selection of exercise variants by practitioners. The second limitation is related to the use of sEMG analysis without a biomechanics analysis that could have assessed moment arms and joint torques, therefore future research is needed to verify how different deadlift exercises influence in other kinetic and kinematic variables. Third, this study did not assess the effect on sEMG, force, and biomechanics parameters of using different inertial loads, which could be investigated in the future. Lastly, this study enrolled a sample of healthy team-sport athletes (male and female), therefore our findings are specific for such a population, but they should be used with caution when professional or elite athletes are tested or trained using flywheel resistance deadlift exercises due to their different physical and competitive levels.

## 5. Conclusions

This study is the first to analyze and compare force production and sEMG of the biceps femoris, semitendinosus, and gluteus medius muscles during the flywheel deadlift exercise in different force vectors and during unilateral and bilateral conditions in team-sport athletes. We concluded that vertical flywheel deadlift variants generated the highest mean and peak force values and were the only variants that demonstrated the achievement of eccentric overload. In particular, the Bi + Ver variant registered the highest average and eccentric peak force production compared with the rest of the exercises. Similarly, the Uni + Ver variant showed higher mean and peak force production than the Uni + Hor variant. In addition, the Uni + Ver flywheel deadlift variant registered the highest relative and absolute sEMG values. However, significant differences were only observed with respect to the Uni + Hor variant in BF_Prox_ and ST sEMG_peak_, and BF_Med_ and ST sEMG_RMS_, and with respect to the Bi + Hor variant in BF_Med_, ST, and GM sEMG_peak_. Although there were no significant differences in sEMG outcomes between the two vertically loaded variants, the use of the Uni + Ver exercise may offer a more demanding unilateral sport-specific (i.e., movement that mimics a recurring movement pattern) stimuli that may lead to a superior transfer to athletic performance and could be used to reduce muscle asymmetries between limbs. In any case, both vertically loading variants developed higher eccentric force values at long muscle–tendon unit length, where injury mechanisms tend to occurs, such as the late swing during high-speed running. Consequently, these exercises could be used to acutely stimulate lower limbs before competitions or training, or they could be implemented into muscle injury prevention protocols.

## Figures and Tables

**Table 1:** Mean ± SD of force production and sEMG outcomes for each flywheel deadlift variant.

	Bi + Ver	Bi + Hor	Uni + Ver	Uni + Hor
**Force** Average (N)	488 ± 132 ^1,2,3^	263 ± 101 ^3^	303 ± 73 ^3^	187 ± 50
Con Peak (N)	643 ± 212 ^2,3^	696 ± 202 ^2,3^	375 ± 115	326 ± 98
Ecc Peak (N)	756 ± 272 ^1,2,3^	592 ± 163 ^3^	495 ± 117 ^3^	266 ± 72
**Muscle activity** BF_Prox_ sEMG_RMS_ (% max)	32.2 ± 4.9	29.9 ± 7.9	31.7 ± 5.1 ^3^	27.0 ± 3.9
BF_Prox_ sEMG_peak_ (μV)	206 ± 78	177 ± 96	234 ± 79 ^3^	170 ± 93
BF_Med_ sEMG_RMS_ (% max)	31.8 ± 4.2 ^3^	29.2 ± 5.0	33.0 ± 4.8 ^3^	27.0 ± 4.3
BF_Med_ sEMG_peak_ (μV)	206 ± 100 ^1^	113 ± 61	234 ± 96 ^1^	215 ± 109
ST sEMG_RMS_ (% max)	31.0 ± 5.4	31.8 ± 5.0 ^3^	32.5 ± 6.2 ^3^	26.7 ± 5.5
ST sEMG_peak_ (μV)	282 ± 158 ^1^	155 ± 75	315 ± 156 ^1,3^	218 ± 116 ^2^
GM sEMG_RMS_ (% max)	32.0 ± 6.4	30.2 ± 6.8	33.6 ± 6.5	29.9 ± 5.8
GM sEMG_peak_ (μV)	139 ± 142	99 ± 130	200 ± 143 ^1^	160 ± 105

*Note*: Values are means ± SD. Abbreviations: Bi + Hor—horizontally loaded bilateral deadlift exercise; Bi + Ver—vertically loaded bilateral deadlift exercise; BF_Med_—medial biceps femoris; BF_Prox_—proximal biceps femoris; Con—concentric; Ecc—eccentric; sEMG_peak_—peak electromyographic activation compared with maximal activation; GM—gluteus medius; RMS—root mean square normalized electromyographic activation; ST—semitendinosus; Uni + Hor—horizontally loaded unilateral deadlift exercise; Uni + Ver—vertically loaded unilateral deadlift exercise. ^1^ Significant (*p* < 0.05) difference from the Bi + Hor exercise. ^2^ Significant (*p* < 0.05) difference from the Uni + Ver exercise. ^3^ Significant (*p* < 0.05) difference from the Uni + Hor exercise.

## Data Availability

The data presented in this study are available on request from the 420 corresponding author. The data from our study are not publicly available due to ethical restrictions imposed by the Institutional Review Board (IRB). The sensitive nature of the data mandates strict adherence to ethical guidelines to safeguard the welfare of the participants.
